# Rafting toward a depression biomarker

**DOI:** 10.1007/s00406-025-02071-3

**Published:** 2025-08-19

**Authors:** Mark M. Rasenick

**Affiliations:** Psychiatry and Physiology, University ofIllinois College of Medicine, Jesse Brown VAMC, Chicago, IL 60612 USA

**Keywords:** GPCR, G protein, Antidepressant, Llipid raft, cAMP

## Abstract

The journey from the cytoskeleton and G protein signaling to a framework that might provide a simple blood diagnostic for depression as well as a harbinger of individual antidepressant response, has been convoluted, occasionally frustrating, but ultimately rewarding. Herein, I provide a travelogue of sorts on our progress toward this end, and also chart a course toward the goal of providing a simple, quantitative and objective, biomarker-driven assessment tool that reflects the neurobiology of depressed mood.

## Introduction

G protein coupled receptors (GPCRs) represent the lion’s share of targets for drugs in current use for psychiatry. Drugs for depression have been the subject of widespread use since shortly after their introduction in 1957. Despite this nearly seven-decade course of clinical deployment, no clear cellular/molecular action mechanism has been elucidated, either for traditional antidepressants (MAO inhibitors, tricyclic antidepressants or Serotonin uptake inhibitors), atypical compounds or the newer rapid acting compounds (ketamine and, perhaps, psychedelics).

Sadly, although likely fitting with respect to the lack of understanding of therapeutic mechanisms, the etiology of depression also remains a mystery. In fact, there may be several pathways dealing to depression and even the diagnosis may be more a phenotype than a specific disease.

Nonetheless, there appear to be a series of credible and consistent observations in humans, rodents and cultured cells, that track with depression and antidepressant action. While it is not clear whether any of these pathways are causative, or merely “fellow travelers”, dissecting out biological pathways that are consistent with depression severity and antidepressant response may be of enormous benefit, clinically, and that is the subject of this report.

**Fig. 1:**
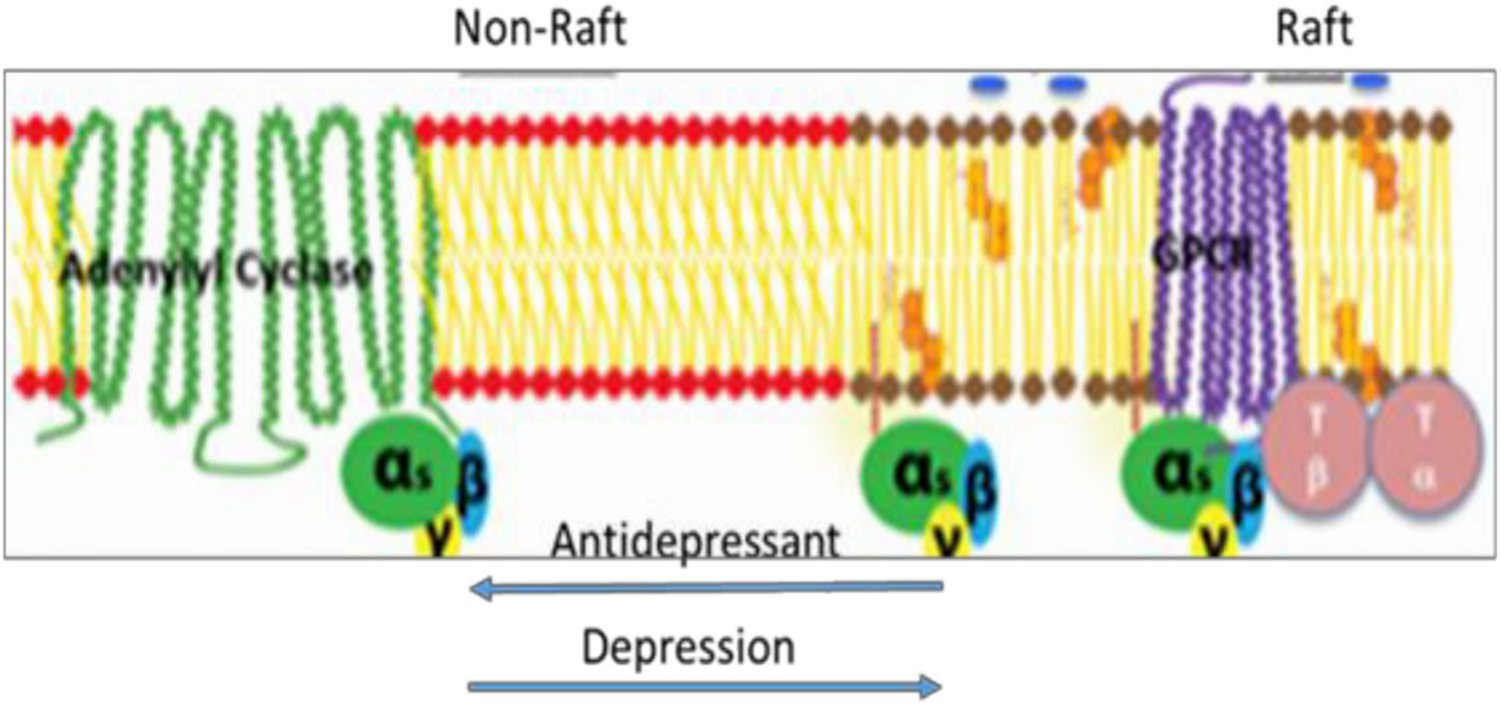
Schematic: Gsα (αs) is normally distributed between non-raft regions of the membrane where it moves freely and promotes neurotransmitter-activated adenylyl-cyclase (AC) activity and a specialized region of the membrane rich in cholesterol (lipid raft), where the movement/adenylyl cyclase activation of Gsα is impaired. During depression, Gsα is enriched in the lipid raft region and it is anchored there by the structural protein tubulin (TαTβ). Antidepressant treatment changes Gsα such that it exits from the raft and moves to the non-raft region where it completes the process of neurotransmitter action by activating the enzyme adenylyl cyclase

More specifically, I wish to provide a scrapbook to illustrate the pathway that has allowed the author to combine interests in the cytoskeleton and G protein signaling with those in the neurobiology of depression to create a viable biomarker for depression and antidepressant response (Fig. 1).

## Signaling is segregated

Plasma membranes are more reminiscent of a crazy quilt than a bedsheet. While cell membranes certainly are comprised of a lipid bilayer, replete with proteins, the distribution of both lipids and proteins is far from uniform, and several regions of the membrane enjoy not only unique lipid distribution but a unique subset of proteins. For the purposes of this treatise, the focus will be on lipid rafts and cellular signaling [18]. Lipid rafts are more concept than single entity, but, as a rule, they provide a rigid, cholesterol-rich, lipid region stocked with specialized proteins that may invaginate (e.g.caveolin) and allow for specific interactions with components of the cytoskeleton. Such interactions facilitate a choreography between the cell surface and subjacent structures and create a series of unique platforms to channel cellular signaling within specific cellular subdivisions [1, 2].

**Fig. 2:**
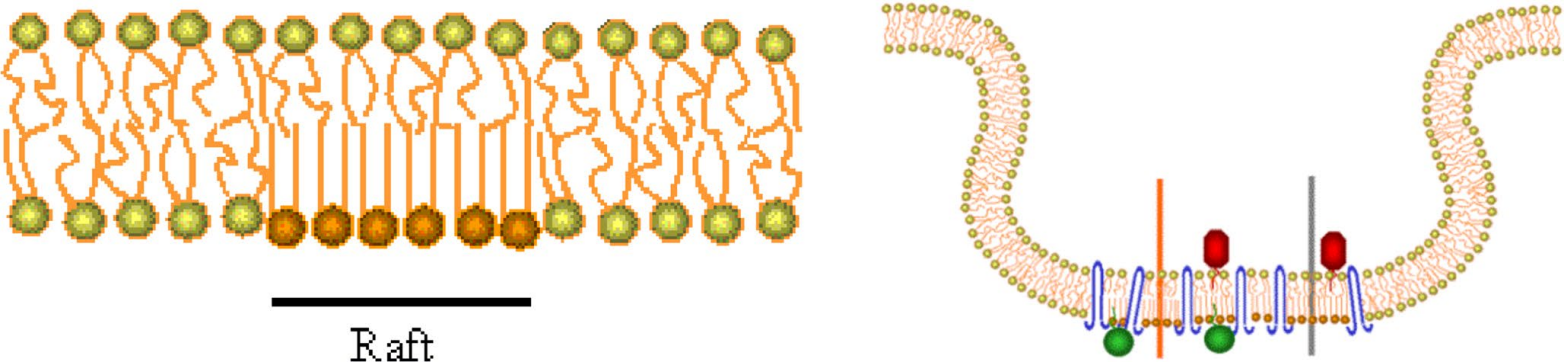
Lipid Rafts. Left: The green balls represent the phospholipid chains that make up much of the plasma membrane. Note that in contrast to cholesterol (brown balls) the phospholipid rich membrane is disordered and fluid. Lipid rafts also associate with cytoskeletal components. Right: Lipid rafts may also contain cavolae (little caves) that contain the protein caveolin (or the similar protein flotillin) and invaginate, further segregating rafts from fluid membrane domains

The narrative below will engage more thoroughly on lipid rafts, membrane compartmentalization and neurotransmitter signaling, but, prior to this, the relevance of cytoskeletal elements to the regulation of neurotransmitter signaling, particularly that through G protein coupled receptors (GPCRs) (Fig. 2).

## Starting out

**Fig. 3:**
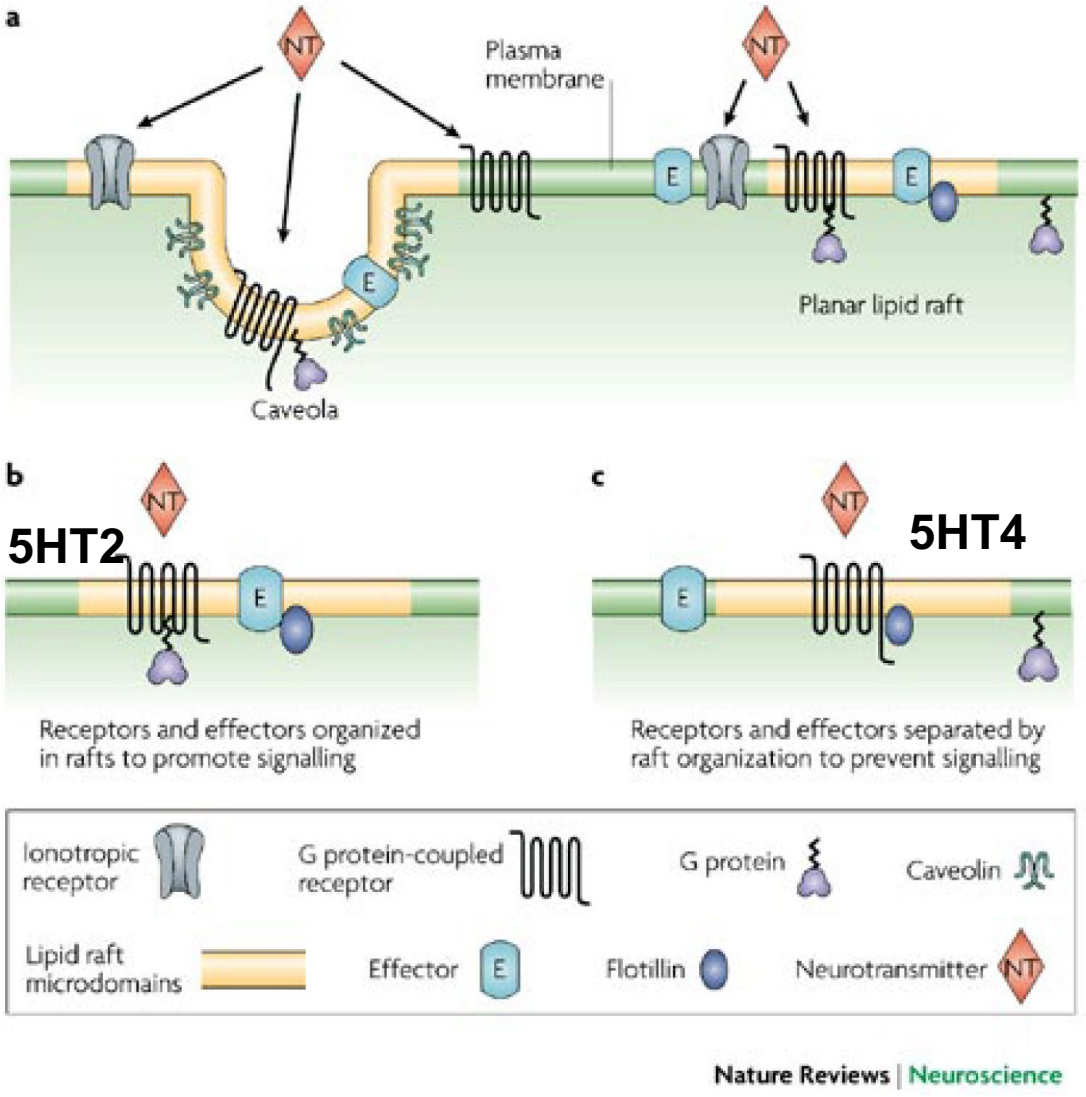
Lipid raft organization of G protein signaling. **a** Lipid rafts (both invaginated (left) and planar (right) have an array of G protein signaling components associated with them. **b** In the case of a 5HT2 receptor, which couples to phospholipase c through Gq, all of the components for this signaling cascade are contained in the lipid raft. Hence, disruption of rafts results in disruption of 5HT2 signaling. **c** For 5HT4, which signals through Gs to activate adenylyl cyclase, the relevant adenylyl cyclase is located outside of rafts. Thus disruption of lipid rafts enhances activation of adenylyl cyclase by GPCR and Gsa. Modified from Allen et al., 2007

My initial project as a postdoctoral fellow was to purify and characterize a protein I had stumbled upon that augmented the stimulation of adenylyl cyclase by fluoride [27]. We had already known that fluoride activates Gsα in the absence of hormone or neurotransmitter, but had yet to learn that it substitutes for the third phosphate when GDP is bound to Gsα in the inactive state. Also unknown to us at time was that the activator is Aluminum Fluoride [34]. Curiously, the protein we had purified (this was before the days of simple protein sequencing) was of a similar size to tubulin (in retrospect, clearly not tubulin due to its basic and hydrophobic properties) and this led to an examination of the specific interaction between tubulin, G proteins (not yet called that) and adenylyl cyclase [28] which continued for several decades (see Schappi et al. [30]: Schappi and Rasenick, [31]) and is still quite relevant to our studies of depression and antidepressant action (Fig. 3).

**Fig. 4:**
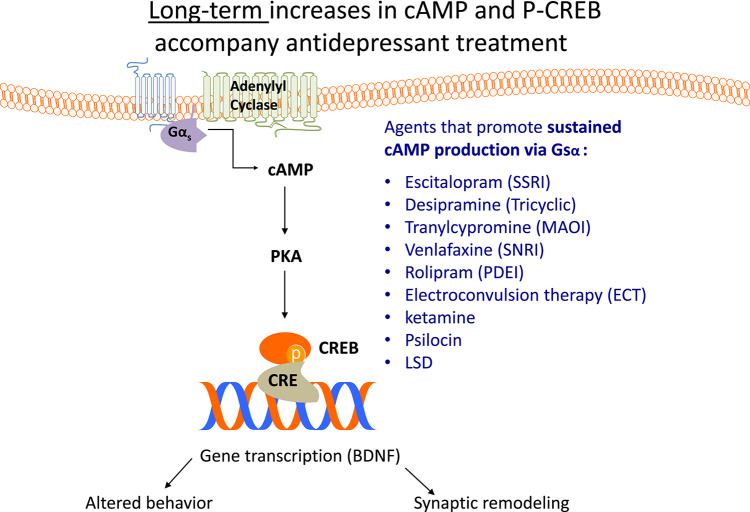
Gsa, cAMP and antidepressant action. The figure illustrates how a large number of antidepressants, of several classes (and ECT in rats) evoke a sustained increase in cAMP, which results in long-term activation (phosphorylation) of the transcription factor, CREB and the subsequent activation of BDNF gene expression and synaptic remodeling. This process is slow in monoaminergic antidepressants and rapid with ketamine

During the course of these studies, I was approached by David Menkes, an MD/PhD student mentored by George Agajhanian, about working with him on a project concerning antidepressants and cAMP signaling. That study [20] showed that sustained (3 week) antidepressant treatment in rats (including electroconvulsive shock) but not brief (1 week) treatment, augmented GTP-dependent adenylyl cyclase activity in multiple brain regions, but not from kidney, liver or heart in those animals (Fig. 4).

## Establishing liberated Gsα as a harbinger of antidepressant action

These studies were refined many times over the next decades, where it became increasingly clear that the cellular locus of antidepressant action resided in increased activation of adenylyl cyclase by Gsα, without any change in the intrinsic activity of that enzyme. A significant breakthrough in these studies was the revelation that we could duplicate animal studies in cultured neural or glial cell lines. Unlike rat studies, where 3 weeks of treatment was required to see an antidepressant response [25], three days were sufficient in C6 glioma cells. While previous studies had suggested increases in the cellular content of Gsα were responsible for the increased cAMP “tone” following antidepressant administration, the cellular models revealed, convincingly, that the amount of Gsα remained stable, but the activation of adenylyl cyclase by Gsα was enhanced [5]. A contemporaneous study with the same model system revealed that, as had been observed in the pioneering rat studies of Sulser and colleagues, antidepressant treatment resulted in desensitization of β adrenergic receptors. However, the cultured cell system revealed that the receptor desensitization was uncoupled, temporally, from the facilitation of Gsα activation of adenylyl cyclase—the former occurring after 24 h of drug exposure while the latter required 72 h [6]. While the bulk of our studies have been done with C6 glioma cells, antidepressants showed similar effects in primary astrocytes. SK N SH neuroblastoma cells, human neural stem cells derived from skin cells converted to pluripotent stem cells, and lymphoblasts [8, 40].

## Moving toward mechanism

**Fig. 5:**
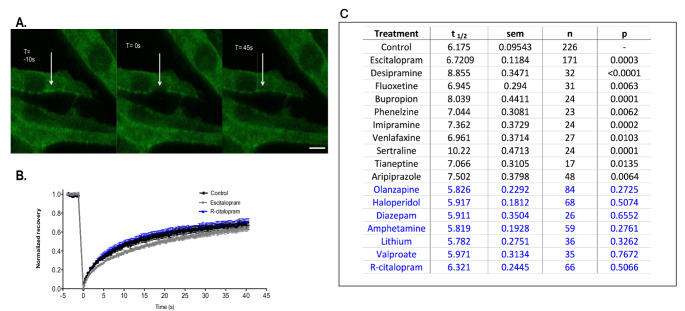
Antidepressant treatment retards Fluorescence Recovery After Photobleaching. GFP-G*α*_s_ recovery after photobleaching is slower after chronic but not acute antidepressant treatment. C6 glioma cells stably expressing GFP-G*α*_s_ were cultured in phenol-red-free DMEM and membrane regions were photobleached. **A** Demonstration of representative photobleaching and recovery of GFP-G*α*_s_. Scale bar represents 10 mm. **B** Demonstration of time course of recovery after photobleaching of control and 10 μM escitalopram or R-citalopram (72 h) -treated cells. Half-time to recovery of GFP-G*α*_s_ is increased after (**c**) chronic (72 h) but not (d) acute (1 h) escitalopram, desipramine, and fluoxetine treatments at 10 μM. Chronic (72 h) R-citalopram had no effect on half-time of recovery. Data were analyzed by one-way ANOVA followed by Tukey's test for *post hoc*multiple comparisons of means (control *vs* treatment, *p < 0.05, **p < 0.01, ***p < 0.001, ****p < 0.0001). Error bars represent SEM. **C** FRAP experiments were performed as described but with various additional antidepressants. All classes of antidepressant increased the half-time to recovery, although the magnitude of this effect varied among drugs, rather than classes. *R*-citalopram had no effect on the membrane mobility of GFP-G*α*_s_. Psychotropics from a variety of classes including stimulants, antipsychotics, and anxiolytics did not alter GFP-G*α*_s_ FRAP recovery time

The addition of Robert Donati to our research group proved fortuitous in hunting for a mechanism by which antidepressant treatment evoked a sustained increase in adenylyl cyclase activity. Dr. Donati’s thesis concerned the membrane protein, caveolin-1 and its contribution to lipid rafts. Lipid rafts are cholesterol-rich structures, associated with the cytoskeleton. Caveolin often joins these raft structures, providing some invagination (caveolae). Studies by Donati, along with Sadamu Toki [12, 37] revealed that sustained (3 weeks in rats, 3 days in cells) translocated Gsα from lipid rafts, resulting in enhanced adenylyl cyclase. Shorter time periods (one day in cells, one week in rats) did not effect Gsa translocation. The process was also antidepressant dose-dependent (Fig. 5).

**Fig. 6:**
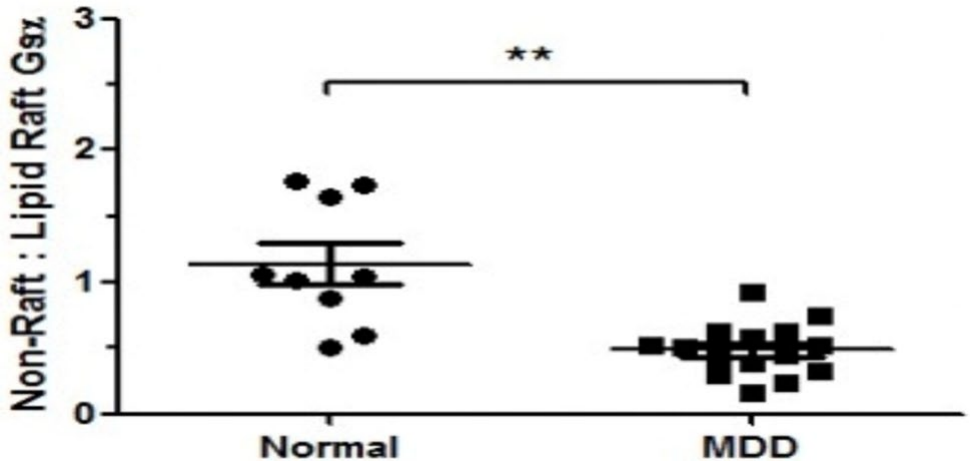
Lipid raft distribution of Gsα in platelets is increased in MDD subjects. Blood was drawn from normal volunteers or patients at the Psychiatric clinic of the Polytechnic Institute of Marche (Ancona, Italy) as part of a lipidomics collaboration with Dr. Massimo Cocchi (see [7]. Fifteen subjects with “moderate depression” were selected. All subjects (including 9 controls) were evaluated for other psychiatric disorders with SCID and those with other diagnoses were excluded. Subjects were either drug-naïve (n = 9) or untreated for 6 weeks (n = 6). Three 8 ml tubes of blood were collected and separated into RBC, leukocyte and platelet fractions, frozen and shipped on dry-ice to UIC, where membrane fractions were prepared and equal amounts of protein extracted, sequentially with Triton X-100 and Triton X-114. Variation in total extracted was about 20% among samples, but there was no significant difference in total Gsα between MDD and control membrane extracts. Samples were dissolved in SDS buffer and subjected to SDS PAGE, immunoblotting, ECL visualization and analysis. These data show a trend similar to that seen with postmortem brain samples [10] with a greater proportion of Gsα from depressed subjects ensconced in lipid rafts. This is also consistent with data showing that Gsα-activated adenylyl cyclase is attenuated in platelets from depressed subjects [17, 21, 22]. Unpaired t-test shows a significant difference between Normal (1.141 ± 0.16 n = 9) and MDD (0.4873 ± 0.051 n = 15) (**p < 0.0001)

While these studies were underway, attempts to develop a mechanistic understanding toward sequestration of Gsα in lipid rafts and the effects of this process on cAMP signaling were carried out. Curiously. genetic or pharmacologic disruption of lipid rafts (caveolin 1 knockout or methyl β cyclodextrin) resulted in augmented GPCR activation of adenylyl cyclase. At the same time, it appeared that activation of phospholipase c through 5HT2A receptors was inhibited by raft disruption. This suggested a clear rationale for the specific effects of antidepressant treatment by Gsα (no other G proteins are affected) and subsequent sustained activation of adenylyl cyclase. It is important to note that antidepressant treatment does not disrupt lipid rafts. In fact, several G proteins are expelled from disrupted lipid rafts, while Gsα exodus is specific to antidepressant treatment (Fig. 6).

## Antidepressants mobilize Gsα

As cellular and rodent studies concerning Gsα and depression progressed, it became increasingly clear that antidepressant treatment resulted in redistribution of Gsα. Initially, this was observed through the modified clustering of a fluorescent Gsα on the cell surface [12, 16]. However, it soon became clear that antidepressant treatment elicited the translocation of Gsα from lipid rafts into non-raft membrane domains, where Gsα showed greater efficiency activating adenylyl cyclase. This was observed both directly through cell fractionation and indirectly by changes in fluorescence recovery after photobleaching (FRAP). We had developed a fluorescent GFP-Gsα fusion protein that had biological and signaling properties identical to the native Gsα (Yu et al. 2002) and were able to create a C6 cell line with constitutive expression of GFP-Gsα. This allowed This cell line, that expressed GFP-Gsα at a level close to that of the endogenous Gsα permitted a study of a large number of compounds. Those latter studies evaluated a large number of antidepressant agents, each of which resulted in retarded FRAP. Mood stabilizers, antipsychotics, stimulants and anxiolytic drugs, many of which are highly lipid soluble, had this effect. Curiously, aripiprazole, an antipsychotic with stand-alone antidepressant properties showed an antidepressant profile in FRAP studies. The retardation of FRAP by antidepressant drug treatment was counterintuitive, as biochemical evidence revealed, clearly, that Antidepressant treatment ushered Gsα from raft domains into the less ordered, fluid membrane domains. This conundrum was resolved when it became clear that Gsα molecules exiting lipid rafts became associated with adenylyl cyclase, which, due to its 12 transmembrane domains, moves very slowly across the plane of the plasma membrane [9]. Later studies showed ketamine had a similar effect on GFP-Gsα FRAP, except that 15 min treatment was required to see the effect (which was short-lived compared with monoaminergic antidepressants [32, 39].

## Incorporation of antidepressants into lipid rafts

**Fig. 7:**
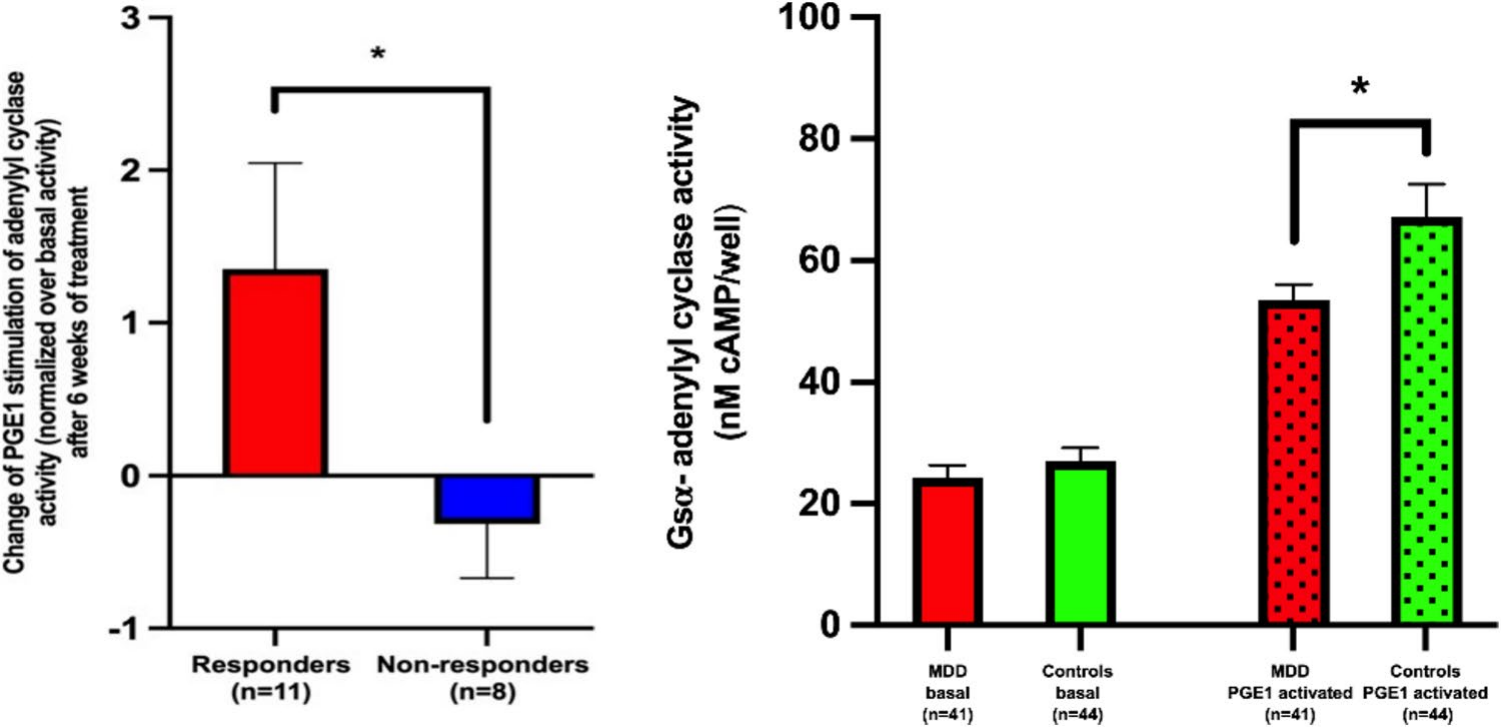
Gsα-activated adenylyl cyclase is attenuated in depression and this is alleviated by successful antidepressant treatment. Platelets were collected from normal or MDD subjects. Cellular membrane fractions were isolated and assayed for cAMP by Alpha Screen per manufacturer’s instructions (Perkin Elmer, MA). (Right), Platelets from depressed subjects at week 0 (pre-treatment) show decreased Gsα- activated adenylyl cyclase without effect on basal activity and (Left) week 8 (post-treatment) recovery correlates with treatment success. PGE1-stimulated adenylyl cyclase values were normalized to the unstimulated/basal value on the right. More complete data, including those for individual subjects are available in [38]. Thus, even though responders and non-responders were exposed to drug, only responders showed increased Gsα-activated adenylyl cyclase. Plasma concentrations of antidepressant were not measured for this study

These studies suggested that, in order for antidepressant drugs to achieve their cellular effects, the drugs themselves should sort into lipid raft fractions. Results from Rainer Rupprecht and his colleagues were certainly consistent with this notion [13] along with the presence of selected ionotropic receptors in lipid rafts [23]. A mass spectrometry study revealed that, while a wide variety of psychotropic drugs bound rapidly to C6 cell plasma membranes, antidepressants alone amongst these compounds became enriched in lipid raft fractions over a three-day period of exposure [14]. This gradual enrichment of antidepressant drug in lipid rafts paralleled the hysteresis seen in augmenting Gsα-activated adenylyl cyclase. The migration of antidepressant compounds into rafts was also stereoselective, as r-citalopram, the clinically ineffective enantiomer of citalopram, did not sort into lipid rafts while escitalopram did migrate effectively. Similar results were seen for the ability of escitalopram, but not r-citalopram to translocate Gsα from lipid rafts [41]. The mood stabilizers, lithium and valproate did not concentrate in rafts nor did these compounds translocate Gsα from lipid rafts [11] (Fig. 7).

## cAMP: causative or a fellow traveler?

cAMP has long been implicated in antidepressant action. Augmented adenylyl cyclase activity as a result of Gsα being more available to adenylyl cyclase results in a sustained increase in cAMP “tone”, evidenced by sustained elevation of phosphorylated CREB and cellular sequelae including increased BDNF production and synaptogenesis [3, 19, 35]. While many of the observations contributing to this have been made in rodents [36], studies by Innis, Zarate and colleagues using ^11^C Rolipram to measure cAMP have shown that depressed subjects reveal lower brain cAMP than healthy controls, and those treated successfully with antidepressants return to baseline cAMP. Subjects who remained depressed, as indicated by both multiple rating scales, showed no rebound in cAMP. Curiously, despite the likelihood that there is some regional specialization to depression, these cAMP changes were widely distributed throughout the brain [15].

Certainly, direct manipulation of cAMP has been a target for antidepressant therapy. The PDE4 inhibitor, rolipram, has antidepressant activity in rodents, but the emetic qualities of this drug have prevented it from commercial adoption. [24].

## Postmortem studies consistent with rodent and cellular observations

We had predicted that, in depression, Gsα was more likely to be ensconsed in lipid rafts, and this was verified by an initial postmortem study [10]. In that study, we saw two brain regions, prefrontal cortex (BA9) and cerebellum with a greater proportion of their Gsα in lipid rafts. The total Gsα was not affected by depression and the three other G proteins (Gi, Go and Gq did not see altered expression or raft distribution in samples from depressed subjects vs controls. Curiously, we had expected cerebellum to be unaffected by depression, yet the data proved otherwise. Analysis of extant literature at the time showed that cerebellum was affected in morphometric studies of depression, suggesting that our “errant” results were actually consistent with the biology of depression in humans [10]. It is noteworthy that the heretofore-mentioned ^11^C rolipram PET imaging studies showed no regional bias [15].

In the [10] study, all depressed subjects had completed suicide. In a more recent study, samples from depressed subjects who had died by suicide and those by other causes were included, although there was no difference between the groups [33]. In this study, the extend of tubulin acetylation was examined. While there was no difference between depressed and control subjects in the overall degree of tubulin acetylation, there was a highly significant difference in lipid raft tubulin, the control subjects having a much larger percentage of acetylated tubulin in lipid raft fractions than those from either depressed group. Curiously, both depressed groups showed much less individual variation than did controls, a finding that was also seen in platelets (vide infra). Acetylated tubulin binds Gsα with a lower affinity than non-acetlyated tubulin, so the lesser degree of tubulin acetylation in lipid rafts from depressed subjects is consistent with the greater degree of lipid raft association for Gsα.

## Blood biomarker for depression and antidepressant response

Initial reports that platelets [21] and leukocytes [26] from depressed subjects showed diminished cAMP production in response to PGE1 (platelets) or isoproterenol (lymphocytes) were published nearly four decades ago. These reports posited that depression reduced sensitivity to either PGE1 or β adrenergic agonist in these peripheral tissues. Later studies suggested that signaling molecules on the surface of human leukocytes might cluster, differently, in depression [4]. We revisited these studies more than a decade ago when, along with Massimo Cocci, we tested platelets obtained from Ancona Italy. Unfortunately, there were no numeric values assigned to patients who classified as “moderate to severely depressed”, but when the lipid raft association of Gsα from the depressed and control group was compared, the extent of lipid raft association of Gsα in the depressed subjects was substantially (p < 0.001) greater, and there was no change in the overall level of Gα expression between the two groups. Curiously, as was seen in the postmortem samples, values from the depressed group were clustered more tightly in the depressed group. These values were obtained through immunoblotting, which is a slow process not suitable for processing more than 24 samples at once (even our automated machine cannot process more than 72 samples/day), hence unsuitable for use as a clinical biomarker. Note that more recent studies also impugned lipid raft clustering of serotonin transporters in white blood cells and their variation with depression [29].

Fortunately, our preclinical studies linked, inextricably, the degree of lipid raft association with the GPCR-Gsα activation of adenylyl cyclase. Joining forces with Steve Targum, a psychiatrist with significant expertise in clinical trials, helped to create a commercially viable biomarker. As a proxy for lipid raft association of Gsα, adenylyl cyclase assay is suitable for high throughput, with assays performed in 386 well format and robotic sample handling. When we applied this assay to samples collected for a previous study, we saw that, as in the preclinical studies, lipid-raft biased localization of Gsα matched the attenuated Gsα-activated adenylyl cyclase seen in depression. Note that the intrinsic adenylyl cyclase activity from both depressed and healthy control platelet samples was equivalent, so the comparator is the multiple of Gsα-stimulated adenylyl cyclase/basal activity. This ratio dampens individual variation, and the assay requires only 0.5 µg/protein per assay point, minimizing the amount of platelets required. When samples were assayed for this biomarker, subjects diagnosed with MDD (n = 40) separated from control (n = 45) at p < 0.02. Depressed subjects were treated for depression (clinicians’ choice, citalopram for most) and biomarker values were determined again after 6 weeks. Those subjects showing response (HAM-D decrease of > 50%; n = 11) reverted to health control values, while non-responders (n = 8) showed no change in biomarker values. The effect size was 0.93; [38]. We have just completed a follow up study, which reveals correlation between biomarker values and depression severity and shows that platelets from individual subjects, collected two weeks apart, show very constant biomarker values as long as their depression scales remain constant. [16].

These results suggested that the ratio between basal adenylyl cyclase and PGE1 = activated adenylyl cyclase might prove a reliable biomarker for depressed mood and, given the one-week lifespan of a platelet, serve as an early indicator of the eventual therapeutic success (or failure) in as little as one week (comparing week 0 and week 1 biomarker values). A follow up study showed that the values for this biomarker were remarkably constant, revealing less than 5% variation within a given subject [16]. Will this biomarker replace clinician administered depression scales or self-evaluation? Certainly not anytime soon, but the availability of a quantitative blood biomarker may be the ideal instrument to induce the reluctant subject into treatment under the notion “It’s not just in my head---It’s in my blood.”.

## Conclusion

This chapter describes a journey that started before the term “G-protein” was used to describe the subject of these studies and before SSRIs were in clinical use. Using a combination of studies in rodents to suggest chemical pathways for antidepressant response, we were able to foster a mechanistic understanding of antidepressants using cultured neural and glial cells. The realization that antidepressants targeted lipid rafts and that they translocated Gsα from lipid rafts in relationship to their speed of clinical action allowed us to suggest a common site of action for drugs with antidepressant activity. From there, it was logical to predict that depression was accompanied by a greater proportion of Gsα in lipid rafts, resulting in dampened cAMP signaling. The revelation that this could also be detected in platelets and the development of a high throughput assay permit a biomarker driven platform for quantitative depression diagnosis and early prediction of antidepressant response. On a preclinical level, this biomarker could aid in the development of antidepressant drugs and might also facilitate clinical trials by biologically-driven subject selection. Finally, this technology might even allow the development of a predictive platform using a patient’s own cells to guide treatment selection and personalize therapy.
